# Factors influencing enrolment: A case study from Birth to Twenty, the 1990 birth cohort in Soweto–Johannesburg

**DOI:** 10.1016/j.evalprogplan.2008.12.002

**Published:** 2009-08

**Authors:** Linda M. Richter, Saadhna Panday, Shane A. Norris

**Affiliations:** a Child Youth Family and Social Development, Human Sciences Research Council, Private Bag X07, Dalbridge 4014, South Africa; b MRC Mineral Metabolism Research Unit, Department of Paediatrics, University of the Witwatersrand Medical School, 7 York Road, Parktown, South Africa

**Keywords:** Longitudinal research, Enrolment, Attrition, Participant retention, Developing country, Participant tracking

## Abstract

Longitudinal studies offer significant advantages in rendering data commensurate with the complexity of human development. However, incomplete enrolment and attrition over time can introduce bias. Furthermore, there is a scarcity of evaluative information on cohorts in developing countries. This paper documents various strategies adopted to minimize loss to follow up and describes a retrospective analysis of a small group of families who were missed during initial enrolment and through several subsequent rounds of data collection of the Birth to Twenty (BT20) birth cohort in Soweto–Johannesburg, South Africa that began in 1990. A purposive case study approach was used, and 10 of the 119 families missed at enrolment were interviewed to investigate why these families were not enrolled into the study. The findings demonstrate that high mobility, both within urban areas and between urban and rural areas, are a major challenge for longitudinal studies in densely populated urban areas. In addition, enrolment was also affected by individuals changing their names, largely motivated to facilitate access to employment under *Apartheid*, as well as varying motivations for participating in research. Longitudinal studies in the developing country context must be mindful of the political, social and economic climate that influences enrolment and ongoing cohort maintenance.

## 1 Introduction

A substantial body of research is available on the evaluation of selective attrition and participant retention strategies in longitudinal studies in developed countries. However, the literature is sparse on these issues from a developing country perspective. Birth to Twenty (BT20) is a longitudinal birth cohort study of child health and development in South Africa. Initiated in 1990, it is to date the largest and longest running study of its kind on the African continent. The study aims to track and document the physical and psychosocial development of children in South Africa's largest and most dense urban environment, the Soweto–Johannesburg metropolis, and to utilize research findings to make informed contributions towards an understanding of the issues impacting upon the development of children.

This paper describes the methodology of the Birth to Twenty study with an emphasis on the recruitment, tracking and management of the cohort over the past 15 years, particularly strategies adopted to minimize loss to follow up. In addition, specific attention is given to the initial enrolment phase and to initial under-enrolment of some socio-economic groups of the planned cohort. Given the limited documented experience on setting up and maintaining longitudinal cohorts in developing country contexts, this study aims to provide methodological insights into aspects of longitudinal designs, enrolment and cohort maintenance for future research endeavours, as well as to illustrate contextual factors and population dynamics that influence research protocol design. A purposive case study approach was used, drawing upon a school survey in which 119 families were identified as missed at enrolment. We report findings from interviews of 10 purposely selected families to elucidate reasons why they were not initially enrolled into the study.

## 2 Description of the Birth to Twenty study, enrolment procedures, and cohort and attrition management

### 2.1 Birth to Ten to Twenty

In 1988, Noel Cameron at the University of the Witwatersrand, and Derek Yach at the South African Medical Research Council (MRC) began a birth cohort study in Soweto–Johannesburg in collaboration with Lucy Wagstaff, Linda Richter, Nicky Padayachee, Sharon Fonn and others (Richter, Norris, Pettifor, Yach, & Cameron, 2007). The study aimed to track the growth and development of urban children born in the Soweto–Johannesburg metropolis from before their birth in 1990, to the age of 10 years (Richter, Norris, & De Wet, 2004). Birth to Ten was conceived at a significant time in South Africa's political development, opportune to document the development of South Africa's ‘new’ generation, in tandem with that of the country's transition from *Apartheid* to democracy. A cohort could serve as a microcosm of the South African transformation through the unfolding of a new social and political dispensation. Partnership with the Community Paediatrics Department was critical to facilitate engagement (information sharing and recruitment) with communities and local clinics, Soweto in particular, and the recruitment of local fieldworkers to assist with this endeavour. The inception of the study took place at a particularly difficult time in South African history (1988–1990) as *Apartheid* was in the process of deconstructing. A ‘state of emergency’ was in place in response to the significant political violence between communities and the South African police, as well as in response to political factional fighting between ethnic groups (for example between the Xhosa and Zulu ethnic groups). Suburban Johannesburg was minimally affected by the violence, but townships such as Soweto were in disarray. The fortitude of the Community Paediatric Health Care Workers and, Professor Lucy Wagstaff, together with the good reputation of the Baragwanath Hospital in Soweto, where the majority of births in Soweto took place, enabled recruitment and building a trusting relationship with township communities. Ironically, private health care facilities and White participants, in particular, were far less trusting or interested in participating in the study.

### 2.2 Initial recruitment process and evaluation

A number of pilot studies were conducted to investigate sampling and feasibility. Areas explored included monthly birth rates and seasonality of births, the extent to which routine health service data was reliably and accurately collected, follow-up logistics and potential sources and patterns of attrition. Details of the outcomes of the pilot studies have been published elsewhere (see Richter et al., 2004).

A number of factors that would influence follow-up were identified. Consultation with health services and local authorities revealed that 20% of deliveries could not be located 6 months later due to inaccuracies in contact information provided by the mother. A large proportion of these contacts were falsified to circumvent restrictions on residence and service usage by *Apartheid* legislation. In all likelihood, this group of women came from areas outside of Soweto–Johannesburg and returned to their rural homes after the birth of their child. It was, therefore, estimated that the residential cohort would comprise 80% of all deliveries.

Local regulations in Johannesburg and Soweto require documentary notification of all births. While these logs provided a useful source from which the initial birth cohort could be derived, it was later discovered that many of the notifications existed in duplicate. They were also affected by inaccuracies such as birth date and name spellings (especially given the wide variety of African languages used and the anglicized derivatives thereof). Duplicate cases had to be removed and corrections made for errors in accuracy and consistency (Anderson & Richter, 1994; Richter et al., 2004). Such errors continued to impact on recruitment and cohort management as will be discussed later. Two additional features identified to be potential impediments to follow-up were the high levels of mobility within and out of the study area, and lack of telephones, addresses, post boxes and other reliable information to maintain contact with participants. At the time, much of Soweto did not have street names or house numbers. With the 20% loss estimated to occur as a result of non-resident births, it was nonetheless estimated from pilot study follow up, that 60% of all births and 80% of the residential birth cohort could be reliably followed-up over several years.

Given the findings of the pilot studies, the ‘cohort’ was defined as follows: timing of a singleton birth within a fixed 7 weeks enrolment period – in the first quarter of the year (23rd April to 8 June 1990) – to ensure a close age range, and residence at time of birth and for at least for the first 6 months of the child's life, within the designated area. This was the ‘Soweto–Johannesburg metropolis’, defined as the local councils of Soweto, Johannesburg and Diepmeadow. The area consisted of roughly 200 km^2^ and 3.5 million people.

Because of limited resources in the study, several decisions had to be taken about the optimal way in which recruitment and follow-up would take place. It was decided that public (state) health facilities would be the primary target for enrolment, followed by private facilities (Richter et al., 2004). This decision was motivated by the small percentage of the population who used private facilities (roughly 15%), and the difficulties of accessing them, proportional to the resources that would be required to trace this group. In addition, users of private facilities (composed mainly of White and Indian South Africans) were not representative of the majority of the population in the area (composed of Black and Coloured, of mixed ancestry, South Africans). The decision not to follow up those families who left the study area was also based on resource availability, and the need to limit work to a finite sample area. However, since 2001, increased funding has enabled families to be traced within a further 100 km radius of the original study area.

The degree of success of this recruitment strategy can be evaluated on the basis of sample information and attrition. During the enrolment period, close to five and a half thousand (5449) births were recorded in the Soweto–Johannesburg metropolis. This constituted the birth cohort for the time period within the area parameters. Of this total number, 3273 cases could be verified as living in the study area for at least 6 months after the child's birth (60%). This latter group is referred to as the residential cohort. Cases were excluded on the basis of stillbirths (112) and child deaths (61) during the first week, in addition to those families who moved out of the Soweto–Johannesburg area immediately after the birth of their child. Table 1 outlines the characteristics of the BT20 cohort. Statistical tests have demonstrated no significant differences between the total birth cohort and residential cohort, except with regard to race (Richter et al., 2004).

### 2.3 Data collection waves

While some rounds of data collection contain measures of special interest, the most common themes run throughout all data collection rounds. These include demographic, socio-economic and household information; community, neighborhood and school environments; health and nutrition; child care, supervision and monitoring; growth and physical activity; cognitive development and school performance; social and psychological adjustment; risk behaviours; and a range of anthropometric and physiological measures. Data has been collected at 19 points across the first 16 years of the study—at antenatal, delivery, 6 months, 1 year, 2 years, 3 years, 4 years, 5 years, 7 years, 9/10 years, 11/12 years, twice during years, 13, 14, 15 and 16, as well as through a school survey at age 13 years (Richter et al., 2007).

### 2.4 Cohort management strategy

The study has been successful in minimizing attrition as a result of systematic analysis and management of factors that may affect follow-up, especially during the initial pilot phase and subsequently as the study evolved (Richter et al., 2004). While the effects of some factors on attrition could not be controlled – such as high mobility, unreliable contact points due to lack of telephones, inadequate street naming and numbering in Soweto, and high unemployment rates that reduced the potential of the workplace as a contact point – other factors that were amenable to some form of intervention were targeted. These include maintenance of an accurate and detailed address database; development of trust and co-operation with the cohort by, amongst others, employing fieldworkers from the local community; maintaining confidentiality; compensation for transport costs; minimisation of time intervals between data collection points; frequent contact (via cohort newsletters, events and workshops); media coverage of the study to reinforce motivation for participation; provision of assistance in the form of a toll-free contact number, and referrals for child problems encountered by families. Points of contact for all participants include telephone numbers of workplace, caregivers and friends; home visits; and home- and school-based delivery of letters.

Through a combination of the strategies outlined above, attrition in the study has been kept to a minimum of 2% per annum from the initial residential cohort to the present sample with which BT20 is still in contact after 16 years (70%). The most common cause of attrition across data collection waves has been high levels of mobility within and out of the study area and problems with contact details. In cases of high mobility, address changes increase the difficulty of tracking participants. Currently, BT20 records reflect up to 848 address changes during any single data collection wave. Urban–rural migration (often involving fosterage of children by rural relatives) means that cases are lost to follow up outside of the study area. Some children may subsequently return to the study area when family conditions change, but it is difficult to systematically monitor such return movement.

Relatively high and low socio-economic status families also accounted for a significant proportion of the BT20 sample lost to attrition over the years (Richter et al., 2004). As a result, targeted approaches for cohort maintenance were required. Over and above transport remuneration, BT20 has successfully maintained the cohort through sponsors providing benefits to randomly selected groups of children, including museum and zoo visits, and film and refreshment vouchers. Families from lower SES groups perceive these opportunities to be of greater benefit than families from higher SES groups. The latter are more amenable to perceived benefits in the form of greater status associated with involvement in altruistic causes. BT20 has attempted to address this by giving regular exposure to the study, its findings and activities in newspapers and on radio and television. Participation among White South Africans was also motivated by reinforcing the perception that involvement in research studies of this nature contributes to redressing past inequalities.

### 2.5 Attrition management and retracing lost cases—the BT20 School Survey

In 2002, BT20 drew upon the school environment as a potential source of contact for lost and missing cases. The Children's School Survey was conducted in primary schools in the study area as well as in bordering areas. Questionnaires were distributed to all children who had been born during the 7-week BT20 enrolment period. These were then checked against BT20 records, and classified as cases with which BT20 is in contact (defined as having collected data for the most recent data wave); cases for which BT20 had collected data at some point but with whom contact had been lost; and cases that had never been seen by BT20.

Of the 5488 questionnaires returned (92% response rate), the Children's School Survey was able to identify 2208 BT20 children. Of these, BT20 had been in contact with 1825 cases (83%) in the last 2 years. The remaining 383 cases (17%) had not been interviewed in the last 2 years. These cases were subsequently contacted and re-enrolled into BT20. The main reason for the lapse of these 383 cases was linked to the family moving to a new residence and the fieldwork team being unable to trace the new address. Over 97% of these 383 families consented to be reincorporated into the BT20 cohort.

The 3280 cases not identified as BT20 cases through the School Survey were composed mostly of children who had not been born in the study area. However, a sample of 119 cases was identified as children potentially ‘missed’ by the initial BT20 recruitment and follow-up procedure. These children were born in the study area during the target time period. A few of these families had been identified, but never interviewed. This sample was seen as a potential source of insight into the methodological strengths and weaknesses of the cohort enrolment procedures adopted.

The 119 cases were predominantly White (*n* = 71, 60%), followed by Black (*n* = 32, 27%) families. Male (*n* = 62, 52%) and female (*n* = 57, 48%) children were almost equally distributed. Most learners were in grade six (*n* = 85, 71%) followed by a small percentage (*n* = 18, 15%) in grade seven. Three quarters of fathers (*n* = 85, 79%) and mothers (*n* = 84, 74%) were employed and the majority lived in a house (*n* = 93, 79%) owned by their parents (*n* = 80, 67%). Most families also had access to basic services namely water (*n* = 96, 81%), sanitation (*n* = 100, 86%), and electricity (*n* = 111, 93%). In the main, this was a relatively privileged group.

## 3 Methods

### 3.1 Case study evaluation

An exploratory case study investigation was undertaken to determine the reasons for our failure to identify and recruit children who were born within the geographical and temporal parameters of the initial enrolment exercise. A ‘case’ was defined as a child born in the Soweto–Johannesburg metropolis during the time period April to June 1990 who had never been interviewed for the study and therefore not enrolled. Eight Black families and two White families were purposively selected to participate in the study from the 119 ‘missing cases’ identified through the School Survey. The comparatively greater proportion of White families in the population of ‘missed’ cases (71 White families vs. 32 Black families) was understandable given the initial decision to recruit first from public delivery centres. As a result of the greater proportion of White families accessing private health facilities, this group was initially underrepresented within BT20. Therefore, only two White families were interviewed to confirm our hypothesis that they were missed due to non-enrolment from private health facilities.

Twenty five percent of the Black families (8 out of 32 Black families) were interviewed for the case study evaluation as it was felt that reasons for their exclusion were not as self-evident as those for White cases, and hence could lead to new information with regard to methodology and recruitment. The eight Black families selected for the study were representative of the socio-economic status of the pool of 32 Black families. Due to time and resource constraints none of the participants of mixed ancestry (‘Coloureds’), of which there were 15 ‘missed cases’, were interviewed.

Semi-structured telephonic or face-to-face interviews soliciting qualitative information were conducted with the primary caregiver of the selected cases. Interviews were approximately 60 min in duration and were conducted by a trained research assistant in the language preferred by the participant. Ethical approval for the study was obtained from the Human Research Ethics Committee of the University of Witwatersrand. Active verbal consent (in the case of telephonic interviews) or written consent (for face-to-face interviews) was obtained from participants. They were informed that participation was voluntary and that the confidentiality of their information would be ensured.

A semi-structured qualitative interview guide was developed to assist the researcher to explore areas postulated to account for failure to recruit cases during the initial enrolment phase and at follow-ups. Themes explored included perceptions of BT20 (had they heard about the study, what they knew about the about the study, were they ever approached to be a part of the study); understanding of research and its potential advantages and disadvantages; recruitment procedures (how studies recruit participants); residential and school mobility over the past 12 years; use and location of public or private health care or educational facilities, and name variations. The interviews were recorded and the information was analysed thematically.

## 4 Results

### 4.1 Description of the case study sample

The case study analyses consisted of eight Black families and two White families. Most cases were male (*n* = 7) and four cases each were in grade five and grade six, respectively, followed by two cases in grade seven. Half of the mothers and fathers were unemployed. Most families lived in a house (*n* = 5) that was self-owned (*n* = 5). The majority of cases had access to basic services, namely water (*n* = 9), sanitation (*n* = 9) and electricity (*n* = 9).

### 4.2 Reasons for failure to interview

Of the 10 cases in the sample, 4 cases had been in contact with BT20 recruitment staff at the time of birth but were not interviewed at any subsequent data collection point. Reasons for failure to interview include migration out of the study area, incorrect contact details furnished, disinterest in the study, and failure of field staff to follow-up with some cases (see Table 2).

The remaining six cases had not been identified by the BT20 recruitment staff at the time of birth but were reported on the birth notification list provided by city regulations. Failure to recruit is linked to the situational factors that characterize the sample (see Table 2). In order to cover as wide an area as possible during recruitment, staff targeted public antenatal facilities, birth facilities at clinics and hospitals, and immunization and child health services. Given logistic constraints and the sampling decisions outlined earlier, staff did not regularly monitor private clinics and hospitals, nor facilities outside of the study area. These reasons, together with high mobility, name changes, and mothers not making use of antenatal or immunization services, accounted for the six missed cases.

### 4.3 Factors influencing recruitment and contact at follow-up attempts

#### 4.3.1 Mobility

Mobility played a central role in failure to recruit initially, as well as to contact families at follow-ups. The case study sample reflects the high degree of mobility within and out of the study area. This finding is supported by the high rate of address changes reported during the various data collection waves in BT20. Most of the cases in the sample changed residence between one and three times during the first 2 years of their child's life. Reasons for residential change were motivated by four factors: financial (caregivers moved because they were unable to afford living costs in an area, often moving in with family for assistance), employment (caregivers moved when employers moved, as in the case of domestic workers, or to take up new work opportunities), schooling (caregivers moved to access better schooling opportunities for their children or to decrease the travelling time to school), and relationships (caregivers moved when partner relationships ended).

#### 4.3.2 Name changes

Case study participants were asked about name variations to examine the extent to which name changes influenced initial recruitment. All participants indicated that they were known by the traditional name provided by their family. However, in at least two of the cases (case A and case G), English names rather than traditional names, were used to register the birth of the child as indicated on the original birth cohort list (compiled from birth notification slips). The English name may have been submitted by the mother, or substituted as part of the clinic or hospital protocol. These changes made it harder to link children to the cohort database.

#### 4.3.3 Study perception

All of the participants had a basic understanding of what is meant by voluntary participation in a research study. Only one participant cited personal disadvantages to participation; case D was concerned about invasion of privacy. Perceptions of research ranged from relatively simple notions, such as ‘a study’ (case A) to more complex interpretations such as ‘scientific investigation of a phenomenon through experimentation or other method’ (case I). Concerns mainly revolved around interferences with school and leisure time – ‘maybe it takes too much time from school’ (case E) - and invasion of privacy – ‘I don’t want people looking at my child and measuring things’ (case D).

Responses showed boundary confusion with regards to treatment and research. Participants perceived the study as a potential source of advice on child rearing practices—‘give me advice on what to do for him’ (case A) and ‘show people what the right way to grow…is’ (case F). Most expressed altruistic intentions in that they believed that the involvement of their child could be helpful for children of that age group of children in general—‘others can also benefit’ (case H). In some cases, this altruism was tempered by reciprocity, in that it was usually paired with perceived benefits for their own child. These benefits ranged from monitoring for abnormalities and receiving treatment – ‘they will see what is needed to improve for [child's name] personally’ (case H) - to perceived psychosocial benefits – ‘he can interact with his own age group’ (case C).

## 5 Discussion

Longitudinal cohort studies are traditionally difficult to sustain in terms of the resources necessary to manage and maintain a cohort with sufficient reliability and validity over long periods of time (das Gupta, Arby, Garenne, & Pison, 1997). Bias in sample composition as a result of both recruitment and attrition can significantly affect the validity of findings, as well as the legitimacy of the original hypotheses across the length of the study time period. This paper has reflected on some of the ways in which these concerns have been evaluated, addressed and managed within the parameters of the Birth to Twenty longitudinal birth cohort study. The comparatively low attrition rate over the duration of the study, despite unique contextual challenges experienced in a developing country, serves as evidence of the success of the Birth to Twenty strategies in limiting attrition and managing the cohort. Of particular interest, given the otherwise successful maintenance of the cohort over the course of the study so far, was to examine where and why recruitment was incomplete and what determined the losses that occurred during the enrolment phase.

The case study analysis demonstrates that high rates of circular migration significantly influenced the ability to recruit participants initially and establish contact at follow-up attempts. These patterns of mobility are linked to the historical migrant labour policies that limited the movement of Black South Africans and that consequently propagated a nomadic labour culture. The movements of Black South Africans were controlled through *passes* and permits that prohibited them from residing in urban areas unless gainfully employed there, or from leaving rural areas unless employed in urban areas (Jones, 1993). The spatial boundaries of African households became extended and considerable variability in household roles and functions developed as a result of high individual mobility, conjugal disruption, illegitimacy, desertion and fragmentation of the traditional nuclear and extended African family unit (Murray, 1981). The migrant labour system, together with the creation of so called ‘homelands’, policies of forced removals and influx control, exercised considerable influence in shaping population movements and the composition of urban, semi-urban and rural settlements.

The economic imperatives compelling people to migrate to metropolitan areas (Jooma, 1991) have not changed with the political transformation and as a result, this stream of movement has continued. Recent census figures have confirmed the existence of a highly mobile population with a growth of 10% in the general South African population since 1996, and a 20% increase (the largest) in the Gauteng region (the province in which the BT20 study is located). This growth has been attributed to internal migration from rural areas (Statistics South Africa, 2001). Gauteng, therefore, receives a considerable number of its residents from other parts of the country. Given such patterns of high mobility, the difficulty and extent of resource expenditure in tracking and managing a cohort such as that in Birth to Twenty is understandable, as are obstacles to follow-up in the initial recruitment phase.

Name variations and name changes also pose a challenge to follow-up procedures as was evident in the case study analysis. In fact, previous research conducted in the Birth to Twenty Study has documented the significant part played by differences in the spelling and variation of African names (Anderson & Richter, 1994), as well as through duplicate entries in official database records. Name changes in South Africa are rooted both in cultural tradition (having multiple names) and the legacy of *Apartheid* that ‘forced’ Black South Africans to renounce traditional names for English or Afrikaans substitutes to facilitate employment. If all names are not recorded, it is possible to lose contact with individuals or duplicate their status as participants within the study. This was exacerbated in the case of caregivers; many of them grew up during the *Apartheid* era and hence, have English or Afrikaans variations to their names.

A significant body of research has confirmed the ways in which *Apartheid* legislation attempted to dilute and deny the cultural identity of non-White South Africans (Martin, 2000). Consequently, the post-transformation period has seen attempts to reclaim identity, and naming, as a significant constituent of identity, is one area in which such attempts have occurred. As a result caregivers may have chosen not to report or disclose an earlier English or Afrikaans name. This process complicates cohort management and may have hampered follow-up.

Our findings also demonstrate that reasons for non-participation amongst some of the cases were convenience-related (time, migration and privacy concerns). Previous studies have also cited respondent-perceived inconvenience as a major factor determining non-participation in research (Hayman, Taylor, Pearl, Galland, & Sayers, 2001). In addition, Shavers, Lynch, & Burmeister (2002) reported that abuse and manipulation by the medical fraternity resulted in non-participation of African-Americans in research studies (specifically medical research) and, in fact, engendered pervasive distrust of research endeavours. There are parallels between these findings and the South African context given that, in 1990, South Africa was still an *Apartheid* state. Many of the intended recruits would have had negative personal experiences of the political regime and hence may have been distrustful of a historically White research endeavour. However, the considerable size of the residential sample recruited by BT20 (3273 cases), militates against a significant role for this factor in recruitment and continued participation. The employment of field workers from the local communities and the setting up of a Community Advisory Board, among other strategies, has decreased the potential for distrust between the community and the researchers.

Limited communication infrastructure also hampered recruitment and follow up, especially during the early years of the study. Given the inadequacies and poor quality of the address framework and postal system in South African townships in general at the time, follow-up via mail posed a real challenge. Boys et al. (2003) reported that the use of a range of communication strategies in longitudinal studies including face-to-face contact, follow-up reminders, postcards and postal contact were useful in keeping attrition rates low. These communication methods are in place in BT20 and contribute to the low attrition discussed previously. However, the high rate of mobility within and out of the study area, coupled with postal system deficiencies, made it difficult to maintain regular contact with the cohort.

Telephonic follow-up was also initially difficult due to the small proportion of the sample who owned telephones, lack of work contact numbers due to high unemployment rates and, inconsistencies in home contact details because of high mobility (Richter et al., 2004). To overcome these challenges and ensure the ability of the study to track the cohort, BT20 obtained contact details of relatives and friends as well as makes frequent visits to the recorded home address. The recent increasing availability of mobile telephones has tremendously improved communication with the cohort.

The case study analysis explored study perceptions and motivations for participation in a study such as BT20. Altruism, coupled with some benefits for the child's physical and psychological health was noted. Groves, Cialdini, and Couper (1992) have reviewed a number of possible explanations for decisions relating to research participation. These include societal-level factors, design attributes, characteristics of the sample person, interviewer attributes, and interaction between interviewer and respondent, as well as psychological factors such as compliance and helping tendencies. The decision to participate in BT20 has been shown to vary by virtue of contextual dynamics on all these. A number of factors impacted on the relationship between the study, as a site of knowledge production, and the subjects of research. These included the historic-temporal context of the study at the brink of transformation after an extensive period of oppression, the politicization of race relations and the unequal distribution of educational and economic resources.

## 6 Conclusion

We appreciate that retrospective studies are plagued by memory recall and *post facto* rationalisation, and that there is no guarantee that the discussion with non-recruited participants is an adequate reflection for reason at the time of enrolment. Furthermore, we acknowledge that the sample was only of children enrolled in school and that the school survey would have missed school leavers, children who died, and children who moved out of the province. However, the findings from this survey not only present a process of tracing cohort participants and potentially reducing attrition, but also assist in evaluating and understanding recruitment and maintenance of a birth cohort. Very few long-term studies have documented such evaluations. The cases (two White families and eight Black families) selected for the study were representative of the broader socio-economic status of the pool of missed cases. While other factors may account for the non-participation of the remaining 109 missed families, we believe the case study has identified the critical elements for non-enrolment.

The Birth to Twenty longitudinal study has been successful in recruiting and maintaining a birth cohort in the Soweto–Johannesburg metropolis during a period of rapid transformation in South Africa, as evidenced by the low attrition rates over the duration of the study. This case study investigation revealed the complex and dynamic interplay of broader social, cultural, political and economic factors that influence the pragmatics of the research process. Such insights can be useful in guiding future research in South Africa and other developing country contexts as well as the design, implementation, analysis and interpretation of study results.

While factors such as migration, and name changes are not readily within the control of research studies, what the findings do show is that a thorough assessment of contextual variables that may influence enrolment and maintenance is required. Migration is a product of the political and socio-economic history of South Africa and the African continent and is likely to continue well into the future. In fact, other prospective community studies in South Africa (Tollman, Kark, & Kark, 1997) and on the continent (Van Ginneken, Muller, & Odhiambo, 2007) have had to factor in the effects of migration in the design and interpretation of study findings. What longitudinal studies in similar contexts can do is compensate for the effects of migration during sample size determination, or alternatively factor in the significant costs associated with tracking participants.

Culturally sensitive factors such as name changes are best dealt with through building trusting relationships with the community. Community buy-in through negotiation with leaders and employing local field staff have proven to be effective strategies for cohort maintenance in the BT20 study and other such studies in South Africa (Tollman et al., 1997) and on the African continent (Van Ginneken et al., 2007). Future recruitment exercises and cohort management strategies should also emphasise the realistic benefits and costs of the study to potential participants in order to promote motivations for reciprocity, at the same time as guarding against coercion.

In order to reflect on and improve the cohort retention, including the enrolment of the second generation of participants (the children of BT20 children), this retrospective analysis of missed cases was undertaken. While enrolment is frequently planned at a technical level, as it was in BT20, this analysis demonstrates the importance of taking into account the broader social, political and economic context in which the study takes place. This context creates challenges that need to be recognised in developing the design and methodological procedures for participant enrolment.

## Figures and Tables

**Table 1:** Demographic characteristics of the Birth to Twenty Cohort.

Characteristics	BT20 cohort (*n* = 3273)
Population group	
African	2568 (78%)
White	207 (6%)
Coloured	383 (12%)
Indian	115 (4%)

Residential area	
Soweto/Diepmeadow	2429 (74%)
Suburban Johannesburg	343 (11%)
Former Indian/Coloured areas	432 (13%)
Inner city	69 (2%)

Maternal age	
<17 years	92 (2.8%)
17–19	392 (12.0%)
20–38	2692 (82.2%)
39+	95 (2.9%)
Not known	2 (0.1%)

Marital status	
Single	1787 (54.6%)
Civil/traditional marriage	1057 (32.3%)
Live together	213 (6.5%)
Common law	46 (1.4%)
Divorced/widow	46 (1.4%)
Not known	23 (0.8%)

Gestational age	
<37 weeks	388 (12.0%)
37–41	2772 (85.0%)
42+	11 (0.3%)
Not known	102 (3.0%)

Birth weight	
<1500 g	30 (1.0%)
1500–2499	322 (10.0%)
2500–3999	2826 (86.0%)
4000+	89 (3.0%)
Not known	6 (0.2%)

**Table 2:** Reasons for non-enrolment of cases.

Case	BT20 contact points	Reason for non-enrolment
Contact established with four cases		
A	Immunization	No time for interview at first point of contact. BT20 staff did not follow up.
B	Hospital	Declined participation due to pending migration out of study area.
C	Birth site	Clinic provided incorrect contact details.
D	Immunization	Declined participation due to privacy concerns.

Contact not established with six cases		
E	BT20 site for birth only	Planned to leave the study area after birth. Not recruited into the study. Subsequently, frequent migration for employment reasons. Failure to recruit during follow-up stages.
F	BT20 site for birth and present in study area after birth	BT20 staff failed to recruit at time of birth. Subsequently, frequent migration for employment reasons. Failure to recruit during follow-up stages.
G	BT20 site at four data collection points	Alternative English name used at birth and family moved to the outskirts of study area. Failure to contact at follow-up stages.
H	BT20 site at four data collection points	BT20 staff failed to recruit at time of birth. Family moved to the outskirts of study area. Failure to contact at follow-up stages.
I	–	White family used private facility for birth hence no contact with BT20 staff.
J	–	White family used facility outside the study area for birth hence no contact with BT20 staff.
